# Longitudinal melanonychia: a rare presentation of Bowen's disease^☆^

**DOI:** 10.1016/j.abd.2022.01.018

**Published:** 2023-05-16

**Authors:** Guilherme de Medeiros Holanda, Guilherme Raya Ravelli, Bruno de Carvalho Fantini, Cacilda da Silva Souza

**Affiliations:** a Division of Dermatology, Department of Internal Medicine, Ribeirão Preto Faculty of Medicine, Universidade de São Paulo, Ribeirão Preto, SP, Brazil; b Department of Pathology and Forensic Medicine, Ribeirão Preto Faculty of Medicine, Universidade de São Paulo, Ribeirão Preto, SP, Brazil

Dear Editor,

A 64-year-old female patient has complained of darkening and deformity of the nail of the third left finger for two years. On examination, onychodystrophy, slight subungual thickening, and irregularly pigmented longitudinal melanonychia (LM) measuring 3 mm were observed (Fig. 1A). Dermoscopy of the nail plate showed irregular longitudinal lines with a variety of colors (light and dark brown, black and gray), and hyperkeratosis located centrally on the free edge of the nail plate (Fig. 1A). Intraoperative dermoscopy of the nail bed and matrix showed slightly irregular and variable pigmentation ranging from black to brown (Fig. 1B); absence of Hutchinson's sign and dermoscopic signs of viral verruca. An excisional biopsy of the nail bed was performed with a margin of 2 mm and healing by secondary intention was chosen. Histopathological examination showed intraepithelial proliferation of atypical keratinocytes with loss of polarity (Fig. 2A), and nail plate with focal hyperkeratosis. Immunohistochemistry was positive for AE1/AE3 (pan-cytokeratin; Fig. 2B), and negative for HMB45 and S100 protein, disclosing the epithelial and non-melanocytic nature of the dysplastic cells, compatible with Bowen's disease (BD).

**Figure 1:**
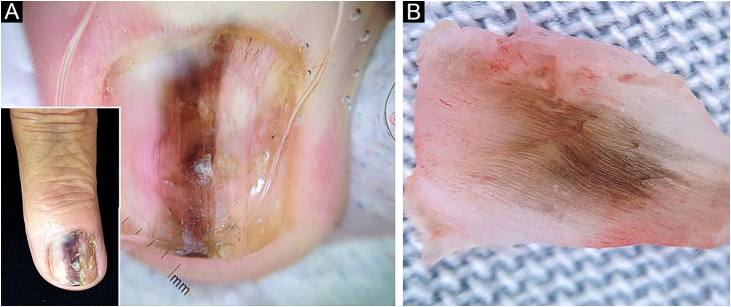
Longitudinal melanonychia of the nail plate: (A) Onychodystrophy associated with irregular and heterogeneous pigmentation in a 3-mm wide band on the third left finger in the miniature; dermoscopy of the nail plate showing irregular longitudinal light and dark brown, black and gray lines. (B) Intraoperative dermoscopy showing variation in pigmentation (brown, gray and black) of the slightly irregular lines

**Figure 2:**
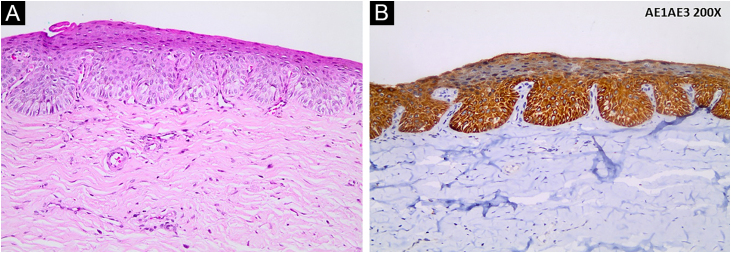
Histopathology and immunohistochemistry findings: (A) Intraepidermal lesion with proliferation of atypical keratinocytes with loss of polarity (Hematoxylin & eosin, ×200). (B) Positive immunohistochemistry for AE1/AE3 (Pan-cytokeratin, ×200)

Ungueal BD, or squamous cell carcinoma (SCC) *in situ*, has a variable record of its frequency, possibly due to failure in its recognition or underreporting. On the other hand, SCC is the most common neoplasm of the nail apparatus, often with late diagnosis. Typically, ungueal BD presents as subungual hyperkeratosis or a verrucous lesion of the nail plate or bed; with periungual erythema and paronychia associated with crusts, ulcerations, or fissures, onychocryptosis, and/or nail dystrophy, and rarely with LM.^1^ It occurs most often in middle-aged men between 50 and 69 years of age. It is usually asymptomatic and grows slowly for years or decades before developing into an invasive SCC. Exposure to ultraviolet radiation and arsenic, immunosuppression, and human papillomavirus (HPV) infection are considered risk factors.^2^, ^3^ Associations, particularly with HPV-56,^4^ seborrheic keratosis and solar lentigo are suggested causes for pigmented BD.^5^ Of 1712 analyzed BD cases, only 90 cases (5.25%) were pigmented BD. Of these, 29% occurred in sites not exposed to the sun, such as genital and intertriginous regions, indicating that other factors, in addition to ultraviolet radiation, may influence the pathogenesis of pigmented BD. In that series, the majority occurred in patients with phototypes I‒III in sun-exposed areas, and only 19% in those with phototypes IV‒VI, who were more likely to have pigmented BD in non exposed areas.^5^

Exogenous pigment deposits (dirt, tobacco), blood, and melanin are frequent causes nail plate and nail bed pigmentation, and melanocytic activation and benign melanocytic nevi are the most common causes of LM in adults and children, respectively. However, about 2/3 of ungueal melanomas present clinically as LM.^2^

Some clinical criteria should raise the index of suspicion of melanoma in acquired LM in adults: the presence of heterogeneous pigmentation in bands or lines of variable colors, fissures or clefts in the nail plate, especially in the distal region (triangular shape), the sudden appearance of nail plate pigmentation, and blurring of the nail fold edges.^2^, ^3^ In nail plate dermoscopy, the main criteria for suspicion are i) nail dystrophy; ii) Presence of gray or black color together with irregular brown pigmentation, iii) Granular pigmentation; iv) And/or involvement of more than 2/3 of the nail plate.^6^

Digital pigmented BD mimics melanoma and may show a chaotic pattern on dermoscopy: atypical parallel pattern of grooves and ridges, and a chaotic pattern with segmental radial lines suggestive of melanoma, associated with other dermoscopic characteristics suggestive of BD, such as squamous surface and linear arrangement of dotted vessels.^7^

LM dermoscopy is limited to the observation of the distribution of pigment deposited in the nail plate, and the underlying lesions can be misinterpreted, which is why intraoperative dermoscopy becomes relevant. Hirata et al. defined four patterns of intraoperative dermoscopy of the nail matrix and nail bed in LM: regular gray, regular brown, regular brown with globules or spots, and irregular pattern.^8^ The irregular pattern was considered the most frequently associated with melanoma,^9^ as seen in the present case.

Among dermoscopy findings, hyperkeratosis located on the free edge of the nail plate was the criterion significantly associated with subungual SCC.^10^ In onychomatricoma, however, the criteria of non-parallel or diffuse edges of nail lesions seem to favor the diagnosis of subungual SCC, among other less specific ones, such as splinter hemorrhages, parallel longitudinal white lines, and nail thickening.^10^

Despite the clinical and dermoscopic findings of the nail plate, and intraoperative dermoscopic findings of the nail bed and matrix raising the suspicion of melanoma, histopathological evaluation did not show proliferation of atypical melanocytes, confirmed by negative immunohistochemistry for HMB45 and S100 protein. Positive immunohistochemistry for AE1/AE3, an antibody against human epidermal keratins, therefore, a marker of normal epithelial cells, carcinomas, and other tumors with epithelial differentiation such as BD, revealed the epithelial nature of the neoplasm in the present case.

Pigmented BD of the nail apparatus is a rarely diagnosed neoplasia and should be considered in the differential diagnosis of LM, particularly in the differential diagnosis with nail melanoma.^1^, ^2^, ^3^, ^4^, ^5^ Changes adjacent to the LM, such as hyperkeratotic areas of the nail plate, should be observed carefully and may help to identify keratinocyte lesions.

A careful evaluation and management of patients with nail pigmentation, taking into account clinical features, dermoscopy of the nail plate, and intraoperative examination of the nail matrix and nail bed can help rule out common causes of nail pigmentation; however, the histopathological examination still remains the reference to confirm the diagnosis of the lesion which is determining LM.

## Financial support

None declared.

## Authors' contributions

Guilherme de Medeiros Holanda: Design and planning of the studied case; collection, analysis and interpretation of data; intellectual participation in the propaedeutic and/or therapeutic conduct of the studied case; critical review of the literature; drafting and editing of the manuscript.

Bruno de Carvalho Fantini: Design and planning of the studied case; collection, analysis and interpretation of data; intellectual participation in the propaedeutic and/or therapeutic conduct of the studied case; approval of the final version of the manuscript.

Guilherme Raya Ravelli: Approval of the final version of the manuscript; intellectual participation in the propaedeutic and/or therapeutic conduct of the studied case.

Cacilda da Silva Souza: Design and planning of the studied case; collection, analysis and interpretation of data; critical review of the literature; drafting and editing of the manuscript; approval of the final version of the manuscript.

## Conflicts of interest

None declared.
